# The role of plant functional groups mediating climate impacts on carbon and biodiversity of alpine grasslands

**DOI:** 10.1038/s41597-022-01559-0

**Published:** 2022-07-28

**Authors:** Vigdis Vandvik, Inge H. J. Althuizen, Francesca Jaroszynska, Linn C. Krüger, Hanna Lee, Deborah E. Goldberg, Kari Klanderud, Siri L. Olsen, Richard J. Telford, Silje A. H. Östman, Sara Busca, Ingrid J. Dahle, Dagmar D. Egelkraut, Sonya R. Geange, Ragnhild Gya, Josh S. Lynn, Eric Meineri, Sherry Young, Aud H. Halbritter

**Affiliations:** 1 Department of Biological Sciences, University of Bergen, Bergen, Norway; 2 Bjerknes Centre for Climate Research, University of Bergen, Bergen, Norway; 3 Norwegian Research Centre, Bjerknes Centre for Climate Research, Bergen, Norway; 4 Office Français de la Biodiversité, Pérols, France; 5 Department of Biology, Norwegian University of Science and Technology, Trondheim, Norway; 6 Department of Ecology and Evolutionary Biology, University of Michigan, Ann Arbor, MI USA; 7 Faculty of Environmental Sciences and Natural Resource Management, Norwegian University of Life Sciences, Ås, Norway; 8 Norwegian Institute for Nature Research, Oslo, Norway; 9 Aix Marseille Univ, Avignon Univ, CNRS, IRD, IMBE, Marseille, France; 10 Simon Fraser University, Vancouver, Canada

**Keywords:** Grassland ecology, Plant ecology, Climate-change ecology, Biogeochemistry, Community ecology

## Abstract

Plant removal experiments allow assessment of the role of biotic interactions among species or functional groups in community assembly and ecosystem functioning. When replicated along climate gradients, they can assess changes in interactions among species or functional groups with climate. Across twelve sites in the Vestland Climate Grid (VCG) spanning 4 °C in growing season temperature and 2000 mm in mean annual precipitation across boreal and alpine regions of Western Norway, we conducted a fully factorial plant functional group removal experiment (graminoids, forbs, bryophytes). Over six years, we recorded biomass removed, soil microclimate, plant community composition and structure, seedling recruitment, ecosystem carbon fluxes, and reflectance in 384 experimental and control plots. The dataset consists of 5,412 biomass records, 360 species-level biomass records, 1,084,970 soil temperature records, 4,771 soil moisture records, 17,181 plant records covering 206 taxa, 16,656 seedling records, 3,696 ecosystem carbon flux measurements, and 1,244 reflectance measurements. The data can be combined with longer-term climate data and plant population, community, ecosystem, and functional trait data collected within the VCG.

## Abstract (summary)

**Table:** 

Measurement(s)	vegetation layer • ecosystem-wide respiration • ecosystem-wide photosynthesis • seedling development stage • temperature of soil • plant functional group biomass • volumetric soil moisture • reflectance spectrum
Technology Type(s)	Visual species identification and cover estimation • Licor gas analyzer • Visual species identification and estimation • ibutton temperature logger • Analytical Balance • SM300 soil mositure probe, Delta-T • GreenSeeker/Normalized Difference Vegetation Index measurements
Factor Type(s)	temperature • precipitation • Plant functional type composition
Sample Characteristic - Organism	Embryophyta
Sample Characteristic - Environment	alpine tundra biome
Sample Characteristic - Location	Vestlandet Region

## Background & Summary

Climate change poses a threat to biodiversity and ecosystem functioning of alpine ecosystems by altering plant, animal and microbial distributions^1,2^, community composition^3^, and food webs^4^; as well as affecting biotic interactions between organisms and functional groups^5–8^. These impacts operate through both direct and indirect pathways. Individual organism’s physiological rates such as photosynthesis and respiration are directly regulated by climatic factors such as temperature and precipitation^9,10^. In turn, these impacts will aggregate to affect ecosystem-level processes such as productivity, decomposition, and carbon and nutrient fluxes in response to climate change^11,12^. Because biodiversity and ecosystem functioning are linked through biotic interactions within and among trophic levels and functional groups^5,13^, indirect impacts of climate, operating on and via these interactions, are also of critical importance. Disentangling the direct and indirect consequences of climate on ecosystems is key to understanding and predicting the full spectrum of biodiversity and functional responses to a changing climate.

Plants are key players in ecosystems: they make up the majority of terrestrial biomass globally, are the main source of photosynthetically fixed carbon, and serve as habitat, food sources, and hosts for other organismal groups, while also competing with them for resources^13–15^. A changing climate will drive predictable shifts in plant species composition; warmer and wetter climates generally favour taller plants, more resource-acquisitive traits, and species that are better competitors for light and nutrients^16^. Previous experiments and observational studies from arctic and alpine systems show that plant functional groups encapsulate much of this variation, and also respond differently to climate and climate change, with graminoids and shrubs typically increasing^17–19^, and bryophytes and lichens decreasing in response to warming^17,18,20,21^. These shifts have knock-on effects on ecosystem productivity, moisture availability, mineralization, and decomposition processes^22,23^.

Removal experiments are a ‘gold standard’ approach for disentangling the roles, interactions, and effects of co-occurring (groups of) plants (see^13,24^). Here we report on a fully factorial removal experiment to assess the roles of and interactions between the major different plant functional groups in alpine grasslands - graminoids, forbs, and bryophytes - in controlling alpine biodiversity, microclimate, and ecosystem functioning. Our semi-natural alpine grassland study system is of high biodiversity and conservation value^25^, yet structurally simple and fine-scaled enough to allow whole-ecosystem (plants and soil community) experimental manipulation. Our experiment was replicated across broad-scale temperature and precipitation gradients in Western Norway in a ‘climate grid’ design that allows disentangling effects of temperature and precipitation change. This is useful, as temperature and precipitation are often confounded, both along elevational gradients and in warming experiments (changes in moisture availability are a side-effect of standard experimental warming approaches, see^26^). We use this study design to disentangle the direct and indirect effects of climate on plant taxonomic and functional diversity, microclimate, and carbon dynamics of alpine grasslands by comparing treatments with plant functional groups removed (direct effects of climate) with treatments with one or more functional groups present (net of direct and indirect climate effects) across sites.

**Fig. 1:**
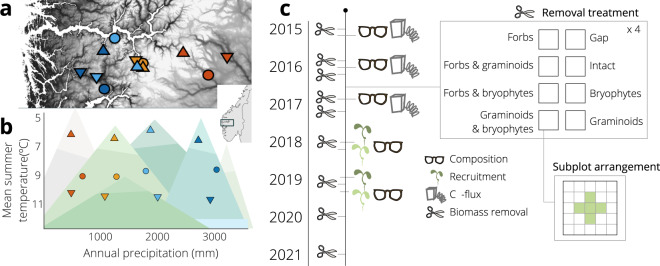
Study area, site selection, experimental design, and field sampling overview for the FunCaB plant functional group experiment. (**a**) Location of the study area and the 12 study sites in Vestland county, Western Norway. (**b**) The Vestland Climate Grid is established across independent broad-scale biogeographic gradients in summer temperature (mean of four warmest months, °C), and annual precipitation (mean annual precipitation, mm). Temperature levels are given as boreal, sub-alpine, and alpine; precipitation levels range from 1 (dry) to 4 (wet). (**c**) Experimental design, with the timeline (species composition recordings [dataset v], seedling recruitment monitoring [vi], ecosystem carbon flux monitoring [vii], and functional group removals [i] indicated, see legend for dataset symbols, for other datasets see text), an overview of the eight factorial removal treatments, and plot layout with subplots used for the community and seedling recording. The removal treatments are described by the functional groups removed from the respective plots, note that in the figure the ‘Intact’ plot refers to the no removal controls whereas the ‘Gap’ are plots with all functional groups removed. For detailed description of treatments and their abbreviations as used in the datasets, see text.

Our experiment is replicated across a macroecological context of twelve calcareous semi-natural grassland sites in south-western Norway (Fig. 1a). The fjords of western Norway offer large variation in temperature and precipitation, and we used this variability to select sites in a ‘climate grid’ where three temperature levels are crossed with four precipitation levels (Fig. 1b). The Vestland Climate Grid (VCG), was established in 2008, and a host of biotic and abiotic data are available from previous research, as detailed below. Within each VCG site, we established the FunCaB plant functional group removal experiment from which we measured carbon and biodiversity responses over 7 years (Fig. 1c). Below, we first describe the site selection and provide basic site information, then outline the experimental design and setup, before we detail variables related to (i) functional group biomass and the removal treatments, (ii) species-level biomass, (iii-iv) soil microclimate, (v) plant community composition, (vi) seedling recruitment, (vii) ecosystem carbon fluxes, and (viii) ecosystem reflectance (Table 1). Note that these data vary in spatial and temporal coverage, as described below. We provide raw and clean datasets with consistent structure and variable naming^27^, and associated code for cleaning and combining data^28^. By documenting and communicating data structures and qualities we aim to facilitate data reuse and combination for new applications in the future.

**Table 1:** Description and location of the datasets in the FunCaB plant functional group experiment.

Dataset	Response variable	Number of observations	Temporal range	Citation information for raw data, clean data, and code
i	Functional group removal	5,412	2015 – 2021	Raw data^27^, clean data^27^, code^28^
ii	Species-level biomass	360	2016	Raw data^27^, clean data^27^, code^28^
iii	Soil temperature	1,084,970	2015 – 2016	Raw data^27^, clean data^27^, code^28^
iv	Soil moisture	4,771	2015 – 2019	Raw data^27^, clean data^27^, code^28^
v	Plant community composition	plant records: 17,181; plant taxa: 206	2015 – 2019	Raw data^27^, clean data^27^, code^28^
vi	Seedling recruitment	16,656	2018 – 2019	Raw data^27^, clean data^27^, code^28^
vii	Ecosystem carbon fluxes	3,696	2015 – 2017	Raw data^27^, clean data^27^, code^28^
viii	Reflectance	1,244	2019, 2021	Raw data^27^, clean data^27^, code^28^

This table summarises information on dataset number, response variable(s), number of observations, temporal range of the data, and location of the primary data, the final published data, and the code for extracting and cleaning data from the primary data.

## Methods

### Data management and workflows

**Fig. 2:**
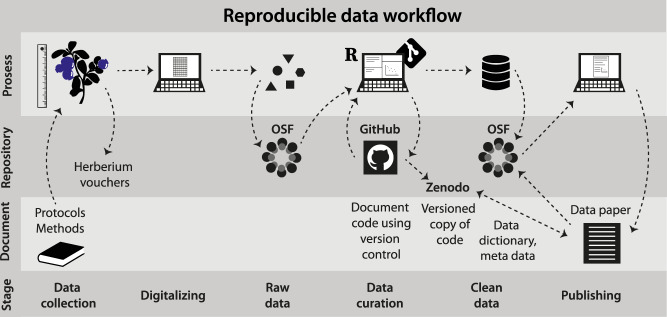
The data collection and management workflow of the FunCaB project. Reproducibility throughout the research process is assured as follows: Experimental design and data collection was based on best-practice community methods and protocols, adapted for the projects’ needs. Measurements were digitalized and the raw data stored in the project Open Science Foundation (OSF) repository before the raw data were cleaned and managed through code-based data curation, with version control secured via GitHub. The clean data are stored at the OSF repository, and a time-stamped version of the code to retrieve and clean data is provided through Zenodo. This data paper describes and documents the data collection and workflow, and describes how to access and use clean data, raw data, and code.

We adopt best-practice approaches for open and reproducible research planning, execution, reporting, and management throughout the project (see e.g.^29–32^). Specifically, we use community-approved standards for experimental design and data collection^29^, and clean and manage the data using a fully scripted and reproducible data workflow, with data and code deposited at open repositories (Fig. 2).

### Research site selection and basic information, and general study setup

#### Site selection

Our study is conducted across the twelve calcareous grassland experimental sites in the Vestland Climate Grid (VCG), in south-western Norway (Fig. 1a). The VCG sites were chosen to fit within a climate grid reflecting a fully factorial design encompassing the major bioclimatic variation in Norway. Potential sites were identified using a combination of topographic maps, geological maps (NGU) and interpolated maps of summer temperature and annual precipitation using the 1960–1990 climate normal (100 m resolution gridded data, met.no; see^33^ and references therein). The three temperature levels (alpine, sub-alpine, boreal) and four levels of precipitation in the climate grid (Fig. 1b) were selected to reflect a difference in mean growing season temperature of ca. 2 °C between three temperature levels (alpine = 6.5 °C, sub-alpine = 8.5 °C, boreal = 10.5 °C mean temperature of the four warmest months of the year) and a difference in mean annual precipitation of 700 mm between four precipitation levels (precipitation levels 1 – 4 representing 700 mm, 1400 mm, 2100 mm, and 2800 mm, respectively). Climate data for the site selection was based on 100-m resolution downscaled data using the 1960–1990 climate normal from met.no. The final sites were selected from approximately 200 potential sites visited and surveyed in the summer of 2008, with selection criteria set to ensure that other factors such as grazing regime and history, bedrock, vegetation type and structure, slope and exposure were kept as constant as possible among the selected sites^34^. Geographical distance between sites is on average 15 km and ranges from 175 km to 650 m.

#### Study system and experimental area selection within sites

At each site, we selected an experimental area of ca. 75 –200 m^2^, targeting a homogeneous and representative part of the target grassland vegetation at large at that site. The experimental areas were placed on southerly-facing slopes, avoiding depressions and concave areas in the landscape and other features such as big rocks or formations that may affect light conditions, hydrology and/or snowdrift. The target vegetation type was forb-rich semi-natural upland grassland vegetation^34,35^, within the plant sociological association Potentillo-Festucetum ovinae tending towards Potentillo-Poligonium vivipara in the alpine sites and Nardo-Agrostion tenuis in some lowland sites^36^. The most common vascular plants across sites, based on sum of covers, are the graminoids *Agrostis capillaris, Festuca rubra, Avenella flexuosa, Anthoxanthum odoratum, and Nardus stricta* and the forbs *Leucantemum vulgare*, *Hypericum maculatum*, *Silene acaulis*, *Alchemilla alpina*, and *Lotus corniculatus*. Common bryophytes are *Pleurotium schreberi*, *Hylocomium splendens*, *Polythricum* spp, *Racomitrium lanuginosum, R. fasciculare*, and *Dicranum* spp. All sites were moderately grazed prior to the study by sheep, cattle, goats, reindeer, deer, moose, and/or horses; and the experimental areas were fenced for the duration of the study to prevent animal and human disturbance of the experimental infrastructure. The fenced area was lightly mowed at the end of each growing season to mimic past grazing pressure and minimize fence effects. For further description of the sites, see^34^ and for access to and further description of site-level data, see^35^.

#### Block and experimental plot setup

**Fig. 3:**
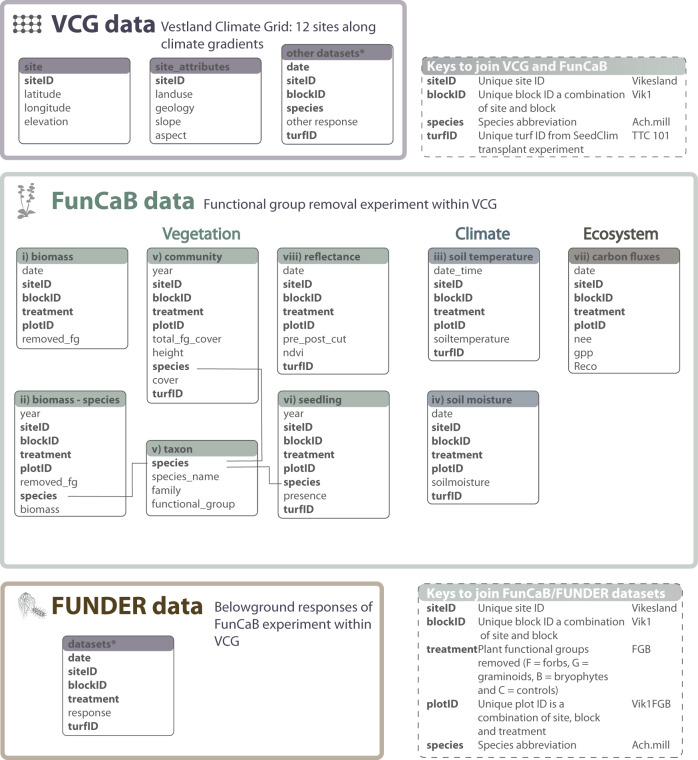
Data structure for the FunCaB functional group removal experiment and associated Vestland Climate Grid (VCG) and FUNDER project data. Within each of the three projects, boxes represent data tables. The FunCaB project data tables include biomass of functional groups removed and forb species-level biomass (datasets i, ii), soil temperature and moisture (datasets iii, iv) plant community composition and the associated taxon table (dataset v), seedling recruitment (dataset vi), ecosystem carbon fluxes (dataset vii) and reflectance (dataset viii). Names of individual data tables are given in the coloured title area, and a selection of the main variables available within tables in the internal lists. For full sets of variables for each FunCaB dataset, see Tables 3–9. The lines linking three of the boxes exemplify links using **species** as keys across tables, note that all bold variables are shared between several tables and can be used as keys to join them. Keys can also be used to link to/from data from other projects in the VCG (for general VCG project keys, see top right hatched outline box, for keys between the FunCaB and FUNDER projects see the bottom right hatched outline box (both including an example value for each variable on the right). The (other) datasets* boxes refer to extensive datasets on plant community composition, cover, biomass, fitness, and reproduction available from previous projects in the VCG^27^ and upcoming datasets in the FUNDER project.

Within these study areas we established four blocks, with a distance between the blocks ranging from one up to (in rare cases) 50 meters. Blocks were selectively placed in homogenous grassland vegetation, avoiding rocks, depressions, and other features as described above. Each block accommodates eight 25 × 25 cm plots, with at least 25 cm between adjacent plots. If a plot contained more than 10% bare rock, shrubs, or other non-grassland features, they were rejected or moved slightly to avoid these features. The plots were permanently marked with four aluminium 10-cm long pipes in the soil in the outer corners of all the 25 × 25 cm treatment plots, ensuring the pipes to fit the corners of a standardized vegetation analysis frame (aluminium frame demarking a 25 × 25 cm inner area, with poles fixed in the corners that fit into the aluminium tubes used for plot demarcation in the field). The upslope left corner tube was marked with a colour-coded waterproof tape. Note that in 31 out of 48 cases (12 sites × 4 blocks), the blocks were located within larger experimental blocks in the VCG sites, and control plots and various block-level data are then shared with other experiments in these larger blocks. Linking keys are described in the FunCaB data dictionaries below (see Fig. 3 and data records iii-vii below). For some datasets, additional plots within blocks were needed. These are described as needed below.

### Background abiotic and biotic data from the Vestland Climate Grid

The Vestland Climate Grid field sites were established in 2008, and from a series of research projects within the grid over the years we have collected a broad range of datasets on the climate and environment, soils, land-use and environment, vegetation, and ecosystems, along with basic descriptive data of the 12 sites, as described in^34^. All these datasets are available from the previous projects through the VCG OSF (Open Science Framework) repository^35^, and the results are presented in associated papers, see for example^34,37–45^. The overall data structure, and the most relevant datasets from the VCG for the FunCaB project is laid out in Fig. 3, and briefly described below. Code to download and link these data to the FunCaB experimental data and sites are provided in the FunCaB github repository^28^ (see R/download_VCG_data).

A new research project, ‘FUNDER - Direct and indirect climate impacts on the biodiversity and FUNctioning of the UNDERground ecosystem’ funded by the Norwegian Research Council KLIMAFORSK programme (project number 315249, 2021 – 2025) will augment the FunCaB experiment with data on the belowground components of the plant-soil ecosystem, including roots, mesofauna, fungi and microbes. These upcoming data will all link with the FunCaB and VCG project based on the given experimental, site and organismal keys, as indicated in Fig. 3.

#### VCG Basic site-level attributes

*Basic* descriptive data on the 12 sites include latitude, longitude, elevation, geology, land-use, soils, and their position within the climate grid (precipitation and temperature levels). These data are described in^34,40^, provided in^35^, and can be downloaded using^28^ (see R/download_VCG_data). For convenience, the climate grid information is also provided in the biomass dataset (see below).

#### VCG Site-level climate data

Temperature was measured continuously at each of the 12 VCG sites at four heights (2 m and 30 cm above ground, at ground level, and 5 cm below ground), soil moisture was measured continuously with two replicate loggers ca. 5 cm below ground, and precipitation was measured at each site during the snow-free season. For these measurements, we used Delta T GP1 loggers (Delta T devices, Cambridge, UK) equipped with two temperature probes, two SM200 moisture sensors which were later replaced as necessary with SM300 and SM150T loggers, and an ARG 100 tipping bucket (EML LTD, North Shields, UK) from 2009 onwards. UTL-3 version 3.0 temperature loggers (GEOTEST AG, Zollikofen, Switzerland) were used for measuring the 2 m and 30 cm temperatures. Soil moisture was measured as the mean of four measurements taken along each side of the turf, several times during the growing season using a Delta T HH2 version 2.3 Moisture Meter with the same probes as for the GP1 logger (SM200, SM150T). These data are described in^34,40^, provided in^35^, and can be downloaded using^28^ (see folder R/download_VCG_data).

#### VCG Soil chemical and structural data

Over the years, various soil chemical variables have been measured at the block level within each of the 12 VCG sites, including soil pH (2009) and % Loss-On-Ignition (2009, 2013), and available N, as sum of N available as NH4-N and NO_3_-N (available N per deployment period, 2010 & 2012). Soil pH was measured after adding 50 ml distilled water to 25 g soil and mixing for two hours. Loss-on-ignition (LOI), was measured by weighing dry soil (105 °C for 24, one hour in a desiccator), and burnt soil (six hours at 550 °C, one hour in the desiccator) and calculating LOI as the (burnt soil mass/dry soil mass) × 100. NH4-N and NO_3_-N were measured using *in-situ* ion exchange resin bags (IERBs) were used to measure the amount of plant-available nutrients in the soil. These data are partially described in^34,40^, and the full data are provided in^35^.

#### VCG Litter decomposition data

Decomposition has been assessed at each of the 12 VCG sites using local plant litter and the Tea Bag Index method (Keuskamp *et al*., 2013). Local litter (dead leaves detached from live plants) was collected at each site in 2013 or 2014, with the specific timing of the collections at each site tuned to ensure that litter was present, not covered by snow, and not decomposed. In practice, this necessitated litter collection after snowmelt in spring in many sites. The litter was washed, dried, and stored in dark, dry, cool conditions. In 2016, five replicate litter bags containing 1 g of graminoid litter were buried at each site, and collected at four points in time after burial (1, 2, 3 and 12 months). Harvested litter bags were cleaned (soil and roots removed), dried for 48 h at 60 °C and weighed. The Tea Bag Index method^46^ was used in 2014, 2015 and 2016 to measure decomposition at all sites of the climate grid. At each site, 10 replicates of each tea type were buried pair-wise, 8 cm below ground and with at least 10 cm between the tea bags. For a couple of sites, the number of replicate tea bag pairs was higher in 2015 (12 replicates at the site Gudmedalen and 16 replicates at Låvisdalen). After collection, adhering soil particles and roots were removed and the tea bags were dried (48 h at 60 °C) and weighed. These data are partially described in^47^, and the full data are provided in^35^ and can be downloaded using^28^ (see folder R/download_VCG_data).

#### VCG Species-level cover, biomass, and performance data

A variety of plant species and community composition, cover, biomass, fitness, and reproductive data exists for the sites and blocks in the VCG from 2008 to 2021. These data are described in e.g^34,37,38,41,43–45,48–50^, and provided in^35^.

#### VCG Site-level plant functional traits

In 2016 and 2017, we measured 11 leaf functional traits that are related to potential physiological growth rates and environmental tolerance of plants, following the standardized protocols in Pérez-Harguindeguy *et al*.^51^: leaf area (LA, cm^2^), leaf thickness (LT, mm), leaf dry matter content (LDMC, g/g), specific leaf area (SLA, cm^2^/g), carbon (C, %), nitrogen (N, %), phosphorus (P, %), carbon nitrogen ratio (C:N), nitrogen phosphorus ratio (N:P), carbon13 isotope ratio (δ13C, ‰), and nitrogen15 isotope ratio (δ15N, ‰). Trait data are available at the site level for species making up at least 80% of the vegetation cover in the control plots at each of the 12 VCG sites. The plants were collected outside of the experimental plots and within a 50 m perimeter from the blocks, and we aimed to collect up to five individuals from each species in each site. To avoid repeated sampling from a single clone, we selected individuals that were visibly separated from other ramets of that species. The sampled plant individuals were labelled, put in plastic bags with moist paper towels, and stored in darkness at 4 °C until processing, which was done as soon as possible and always within 4 days. These data are described in^52^, provided in^35^, and can be downloaded using^28^ (see folder R/download_VCG_data).

### Experimental design

*The functional group removal experiment was designed to* examine the impact of aboveground interactions among the major plant functional groups - graminoids, forbs and bryophytes - on the performance and functioning of other components of the vegetation and ecosystem. The experiment consists of eight 25 × 25 cm plots per site and block, with a fully factorial combination of removals of three plant functional groups, with treatments randomized within each block. The general experimental design, with the different removal treatments detailed, are provided as an insert to the timeline in Fig. 1c. The functional groups are delineated and abbreviated in the various datasets as follows: G = graminoids (including grasses, sedges and rushes), F = forbs (including herbaceous forbs, pteridophytes, dwarf-shrubs, and small individuals of trees and shrubs), B = Bryophytes (including mosses, liverworts, and hornworts). Note that all species are also coded by their respective functional group into which they were classified in the FunCaB taxon table. The experimental treatments are coded by functional group removed so that FGB = bare-ground gaps with all plants removed, FB = only graminoids remaining, GB = only forbs remaining, GF = only bryophytes remaining, B = graminoids and forbs remaining, F = bryophytes and graminoids remaining, G = bryophytes and forbs remaining, and C = intact vegetation controls with no vegetation removed. In 2016, four extra control (XC) plots were marked per site for aboveground biomass harvest and ecosystem carbon flux measurements. This sampling regime gave a total of 384 plots in the core FunCaB experiment, plus the additional 48 controls in 2016.

Functional group removals were done once in 2015 (at peak growing season due to late snowmelt), twice per year in 2016 and 2017 (after the spring growth and at peak growing season) and annually from 2018 to 2021 (at peak growing season) as regrowth had declined (see below) and biannual removals were no longer necessary. At each sampling, all above-ground biomass of the relevant plant functional group was removed from each plot as follows: for each plot, all the above-ground parts of the relevant functional group(s) were removed using scissors and tweezers to cut the plants at the ground layer (i.e., the soil-vegetation interface). Roots and other below-ground parts were not removed, and non-target plant functional groups and litter were left intact.

### Species identification, taxonomy, and flora

All vascular plant species were identified to the species level in the field, with nomenclature following Lid and Lid^53^. Exceptions are sterile specimens of species that are not possible to identify without reproductive parts, and where flowers are either too rare or individuals too short-lived for comparisons of the position of individuals within the plots over years to be used to ascertain identifications (For example, *Alchemilla* spp. excluding *A. alpina*, and the annual *Euphrasia* spp.). Species identifications were confirmed by comparing records over time as described below. All unidentified specimens are included and flagged in the dataset, as described in Data Records below. The full taxon names are provided in the taxon table on OSF (Fig. 3).

### Dataset collection methods

#### Datasets (i–ii): Biomass and functional group removal

As described above, functional group removals were done once in 2015 at peak growing season, and twice per year in 2016 and 2017 (after the spring growth; at peak growing season) and annually at peak growing season from 2018 to 2021. For each removal plot and occasion, a picture was taken of the plot pre-removal, the biomass to be removed was collected in separate pre-marked paper bags for each functional group (graminoids, forbs and bryophytes), and a picture was taken post-removal. The collected biomass was then dried at 60 °C for 48 hours and weighed to the nearest 0.01 g (Model LPG-1002, VWR). From the four extra control (XC) plots in 2016, total above-ground biomass as well as litter (defined as dead biomass detached from live plants, see^28^) was collected at peak growing season. From these plots, biomass was sorted into functional groups as described above, except the forb functional group, which was sorted into species. The graminoid and bryophyte functional groups, each forb species, and litter were individually dried and weighed as described above. The data is available as (i) a biomass dataset, consisting of the removed biomass per plot, date, removal treatment, and functional group for all treatment plots, and the total biomass per functional group plus litter for the extra control plots in 2016, and (ii) a species-level forb biomass dataset from the extra control plots in 2016 (Fig. 3, Table 1).

#### Datasets (iii-iv) – Soil microclimate

We measured soil temperature 3–5 cm below the soil surface for each plot using iButton temperature sensors (DS1922L, Manufacturer reports temperature accuracy of ±0.5 °C, Maxim Integrated INC., San Jose, CA, USA). The data are reported with a resolution of 0.0625 at 140 min intervals from June 2015 to July 2016. We measured soil moisture as volumetric soil moisture; expressed as % water volume per soil volume ((m^3^ water /m^3^ soil) × 100). These measurements were done c. 3–5 times during the growing seasons from 2015–2019, usually in connection with the flux and vegetation measurements, by taking the average of four measurements, one at each side of each plot (SM300, Manufacturer reports accuracy ±2.5% vol over 0 to 50% vol and 0–60 °C, Delta-T Devices, Cambridge, UK). The data is available as (iii) temperature and (iv) volumetric soil moisture % per plot and time point (temperature) or date (moisture) (Fig. 3, Table 1).

#### Dataset (v): Vascular plant community composition and vegetation structure

We recorded the full vascular plant species composition of all experimental plots in 2015 (pre treatment), and the control plots plus the extra control plots in 2016. In 2017, 2018, and 2019, we recorded the community composition in controls and in the functional groups that remained in the experimental plots according to the plot’s treatment. At each analysis, each plot was sub-divided into 25 5 × 5 cm subplots, using a subplot overlay. We first recorded all species of vascular plants in the central five subplots, (i.e., the central + shaped area of each plot, Fig. 1c) noting the subplot cover of each species present in each of the five subplots (1 – 25% = 1, 26 – 50% = 2, 51 – 75% = 3, >76% = 4). Additionally, we noted if the individual was fertile (records circled if buds, flowers, or fruits were present). The five subplots were recorded and numbered (1-5) by row, and from left to right, starting from the top up-slope subplot. For the entire 25 × 25 cm plot, any additional species not present in one of the central subplots were recorded and their fertility noted. We then visually estimated the percentage cover of each vascular plant species in the whole plot to the nearest 1% and measured vegetation height in mm at four points within the plot. Note that the total coverage in each plot can exceed 100% due to layering of the vegetation. The vascular plant vegetation data is available as percentage cover and fertility status (sterile or fertile) per species per subplot and plot per sampling date, and vegetation height in mm per plot per sampling date (Fig. 3, Table 1).

Other variables that were measured were percentage cover of bryophytes, litter, bare ground, and rock (measured per plot and per subplot) and moss layer depth in mm (mean of 4 measurements/plot), date of analysis, recorder/scribe (if any), and free-text comments. These data are available as % cover, depth in mm, date (year.month.day) and text strings per subplot and /or plot per sampling date (Fig. 3, Table 1).

#### Dataset (vi): Seedling recruitment

The total number of forb seedlings that emerged in the plots was recorded in 2018 and 2019. At peak growing season in 2018 (round 1, July-August, depending on site), all dicotyledonous seedlings were marked with wooden toothpicks and their x and y coordinates in the plot (mm, recorded from the bottom left hand-corner of the plot, Fig. 1c) and tentative species identity noted. Toward the end of the growing season (round 2, August-September, depending on site), each plot was revisited, seedling survival established, and any further seedlings marked. Survival (recorded when a seedling was present in subsequent surveys; recorded as mortality if absent) and new seedling emergence were followed up in the same manner in 2019 (rounds 3 and 4, respectively). Species identification was (re)assessed at all censuses and corrected if needed as the seedlings grew and identification uncertainty decreased. New seedlings were differentiated from emergent clonal ramets by looking for cotyledons or signs of above- or below-ground ramet connections. These data are available as talleys of seedlings, each with a status (dead or alive) and species identity (or NA when not identifiable), per subplot and /or plot per sampling round (Fig. 3, Table 1).

#### Dataset (vii): Ecosystem carbon flux data and flux calculations

##### Carbon flux measurements

Ecosystem CO_2_ fluxes were measured to estimate net ecosystem exchange (NEE), ecosystem respiration (Reco) and gross primary production (GPP). The dataset covers the years 2015, 2016 and 2017, and individual plots have multiple measurements for ecosystem carbon flux per year as detailed below. At peak growing season in 2015, a median of 2 sets of paired carbon flux measurements were measured pre-removal for all plots, where a paired set consist of a light and a dark flux measurement of an individual plot. In 2016, a median of 8 sets of paired measurements were made for all control plots, and a median of 7 for the 4 extra controls (see experimental design above). In the data files, some additional measurements exist for other experiments in the VCG sites (a median of 7 paired sets of measurements for controls (TTC) and graminoid removal plots (RTC), see^42^ for a presentation of this experiment and^35^ for technical details). In 2017, a median of 5 paired sets of measurements were made for all treated plots in nine of the sites, excluding the second wettest precipitation level (sites Gudmedalen, Rambera, and Arhelleren). These measurements were made ca. 1 week after the first round of plant functional group removals in that season.

At each sampling occasion, a clear chamber (25 × 25 × 40 cm) equipped with two fans for air circulation and connected to an infrared gas analyzer (Li-840; Manufacturer reports accuracy within 1.5% of the reading value; LI-COR Biosciences, Lincoln, NE, USA) was used to measure CO_2_ fluxes at all plots. To prevent cutting of roots and disruption of water flow in the plots by installing collars, we instead attached a windshield to the bottom of the chamber and weighed it down on the ground by a heavy chain to prevent wind-air mixing. At each sampling occasion we made paired measurements of fluxes under light and dark conditions, covering the chamber with a fitted light-excluding cover for the dark measurements.

NEE was estimated from measurements of CO_2_ flux under ambient light and dark conditions: NEElight = GPP - Reco, NEEdark = (-) Reco. We define NEE such that negative values reflect CO_2_ uptake in the ecosystem, and positive values reflect CO_2_ release from the ecosystem to the atmosphere. For each measurement, CO_2_ concentration was recorded at 5 s intervals over a period of 90–120 s. NEE was calculated from the temporal change of CO_2_ concentration within the closed chamber according to the following formula:

$$NEE=\frac{\delta C{O}_{2}}{\delta t}\times \frac{PV}{R\times A\times (T+273.15)}$$

where $\delta \frac{C{O}_{2}}{\delta t}$ is the slope of the CO_2_ concentration against time (µmol mol^−1^ s^−1^), P is the atmospheric pressure (kPa), R is the gasconstante (8.314 kPa m3 K^−1^ mol^−1^), T is the air temperature inside the chamber (°C), V is the chamber volume (m^3^) and A is the surface area (m^2^).

Light intensity was measured as photosynthetically active radiation (PAR, µmol m^−2^ s^−1^) using a quantum sensor (Li-190; Manufacturer reports absolute calibration accuracy of ±5%; LI-COR Biosciences, Lincoln, NE, USA) placed inside the chamber. Temperature inside the chamber was measured using an iButton temperature logger (DS1922L, Manufacturer reports temperature accuracy of ±0.5 °C, Maxim Integrated, San Jose, CA, USA). Volumetric soil moisture content (m^3^ water/m^3^ soil) × 100 was measured by calculating the average of four measurements with a soil moisture sensor (SM300, Manufacturer reports moisture accuracy of ±2.5%, Delta-T Devices, Cambridge, UK), taken at each side of a plot.

##### Data management and calculations

Data from the LiCOR data logger and iButton was downloaded in the field and stored. The information from the field data sheets (metadata of CO_2_ measurements and plot soil moisture) was manually entered into digital worksheets, manually proof-read and stored. Data from the data logger (PAR and CO_2_) and the iButton temperature logger were linked based on information from the field data sheets. All measurements were first visually evaluated for quality and only measurements that showed a consistent linear relationship between CO_2_ over a time for a period of at least 60 s were used for NEE calculations. A second inclusion criterion was that this relationship had R^2^ ≤ 0.2 or R^2^ ≥ 0.8 for NEE measurement in light conditions and R^2^ ≥ 0.8 for NEE dark measurements (R_eco_). Measurements of NEE in light conditions with R^2^ ≤ 0.2 ensures representation of measurements with equal rates for R_eco_ and GPP. Third, paired measurements that were more than 2 h apart were excluded. These data are available as raw fluxes and as GPP and R_eco_ per plot per measurement (Fig. 3, Table 1).

#### Dataset (vi): Reflectance

Reflectance measures of Normalized Difference Vegetation Index (NDVI) were taken for each plot during the 2019 (post functional group removal) and 2021 (pre and post removal) field seasons (July-August), using a Trimble Greenseeker Handheld Crop Sensor (Trimble Inc., Sunnydale, CA, USA). As the sensor measures an elliptical plane, two measures perpendicular to each other were taken for each subplot (25 × 25 cm plot), with the centre of each ellipse being the centre of the subplot. Care was taken to ensure that sampling quadrat frames were not within the sensor range when conducting measurements (see methods Dataset ii). Measures of NDVI were taken at 60 cm above the surface where possible. Height was measured perpendicular to the sampled ground surface. These data are available as reflectance per plot per sampling date (Fig. 3, Table 1).

## Data Records

This paper reports on data from a plant functional group removal experiment replicated across the twelve sites in the Vestland Climate Grid along broad-scale bioclimatic gradients in boreal and alpine grasslands in western Norway, conducted from the 2015 growing season onwards. The datasets include the treatment variable, biomass removed, and a number of response variables reflecting microclimate, plant community composition, seedling recruitment, ecosystem carbon fluxes, and vegetation reflectance collected from 2015 (the pre-treatment year) through 2021, with variable number of years of data and temporal resolution between datasets (Fig. 1c, Table 1). Additional information and covariables reflecting site climate and environment, soils, litter decomposition, and plant functional traits in the Vestland Climate Grid can be obtained from the VCG data descriptor^35^ and linked to the FunCaB data using shared site, block, plot, treatment and taxonomic variables as keys (Fig. 3). Code to download and link selected VCG data to the FunCaB experiment is provided in the open FunCaB GitHub repository, and in a time-stamped version on Zenodo^28^.

Data outputs consist of 6 datasets, (i) the functional group biomass removed from all treatment plots from 2015 through 2021, (ii) species-level forb biomass from the extra control plots in 2016, (iii) soil temperature in 2015 and 2016, (iv) soil moisture from 2015 through 2019, (v) species composition and structure of the experimental plots from 2015 thorugh 2019, (vi) seedling recruitment in 2018 and 2019, (vii) ecosystem fluxes from 2015 through 2017, and (viii) reflectance in 2019 and 2021 (Table 1). The structure and relationship between datasets are described in Fig. 3. These data were checked and cleaned according to the procedures described in Fig. 2, see Technical Validation for details, before final data files and associated metadata were produced.

The final data files (see Table 1 for an overview) and all raw data, including raw flux measurements and pictures of the plots pre-and post-removals, are available at the FunCaB Open Science Framework (OSF) data repository^27^. To ensure reproducibility and open workflows, the code necessary to access the raw data and produce cleaned datasets, along with explanations of the various data cleaning steps, issues, and outcomes, are available in the open FunCaB GitHub code repository, in the file R/data_dic/download_clean_data.R, with a time-stamped versioned copy archived in Zenodo^28^. The reader is referred to the code and the detailed coding, data cleaning, and data accuracy comments and the associated raw and cleaned data and metadata tables available in these repositories for detailed information about the data cleaning process. Figure 2 and the Usage Notes section in this paper summarises the data accuracy and data cleaning procedures, see the latter for caveats regarding data quality and our advice on ‘best practice’ data usage.

### Dataset (i): Plant functional group removed and total biomass

**Fig. 4:**
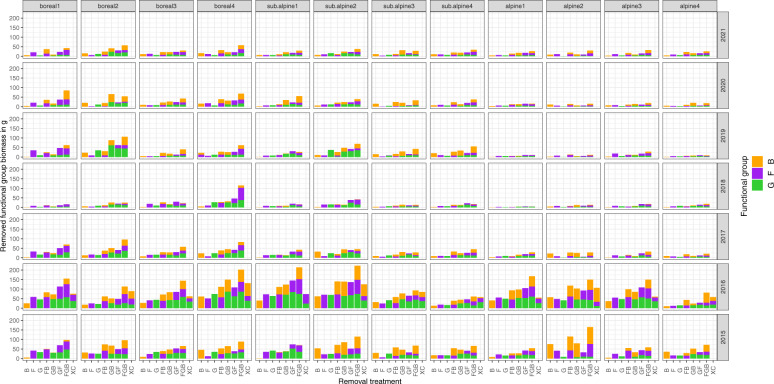
Biomass over time in the FunCaB plant functional group removal experiment. Biomass removed per site, treatment, and plant functional group over time from 384 25 × 25 cm experimental plant functional group removal plots from 2015–2021, and total biomass from 48 extra controls in 2016, at twelve sites in the Vestland Climate Grid, Vestland County, Norway. Sites are coded by vegetation zone (boreal, sub-alpine, alpine) and precipitation level (low = 1 to high = 4). B = bryophytes, F = forbs, G = graminoids. Removal treatments are coded by the plant functional group removed (i.e., in the B treatments, F and G are not removed).

This dataset contains the biomass removed per experimental plot and functional group per year, and the total biomass in the extra controls in 2016 (Tables 1, 2, Fig. 4). For convenience, this dataset also contains basic information on the temperature and precipitation levels in the VCG climate grid (more details available in^35^ and^28^). From the experimental plots we removed a total of 17,839 g biomass over the project period; 5,881 g graminoids, 6,039 g forbs, and 5,918 g bryophytes. Biomass removed decreased over time, 3,742 g, 6,396 g, 1,956 g, 1,095 g, 1,701 g, 1,502 g, and 1,446 g per year from 2015 through 2021, suggesting that the removed functional groups were not able to recover from treatments and gradually were excluded from the treated plots as the experiment progressed. The lowest biomass was removed in 2018, a drought year.

**Table 2:** Data dictionary for the FunCaB functional group-level biomass (dataset i).

Variable name	Description	Variable type	Variable range or levels	Units	How measured
year	Year of sampling	numeric	2015 – 2021	yyyy	defined
date	Date of sampling	date	2015-07-22 – 2021-07-30	yyyy-mm-dd	defined
round	Round of sampling	numeric	1 – 2	count	defined
siteID	Unique site ID	categorical	Alrust-Vikesland		defined
temperature_level	Temperature level with the climate grid	categorical	boreal, sub-alpine, alpine		defined
precipitation_level	Precipitation level within the climate grid (low to high)	categorical	1, 2, 3, 4		defined
blockID	Unique block ID a combination of site and block	categorical	Alr1 – Vik5		defined
plotID	Unique plot ID is a combination of site, block and treatment	categorical	Alr1B – Vik5GF		defined
treatment	Plant functional groups removed, where F = forbs, G = graminoids, B = bryophytes, C = control and XC = extra control	categorical	FGB, FG, FB, GB, G, F, B, C, XC		defined
removed_fg	Removed functional group, where F = forbs, B = bryophytes, G = graminoids. For extra controls also L = litter, P = pteridophytes, LI = lichens, and C = cryptograms	categorical	For experimental plots: F, G, B,For extra controls: F, G, B, L, P, LI, C		defined
functional_group	Removed functional group, including forbs, bryophytes, graminoids. For extra controls also litter, pteridophytes, lichens, and cryptograms	categorical	bryophytes-pteridophytes		defined
biomass	Dry weight of removed functional_group	numeric	0 – 41.31	g	measured
name	Name of data collector	categorical	AB – William		recorded
remark	Remarks	categorical			

Data dictionary with column descriptions for dataset i – the biomass removed from 384 25 × 25 cm experimental plant functional group removal plots from 2015 – 2021, and total biomass in 48 extra controls in 2016, at twelve sites in the Vestland Climate Grid, Vestland County, Norway.

The extra control plots in 2016 contained a total of 1,495 g total above-ground biomass; 357.0 g grasses, 300.0 g forbs, 322.0 g bryophytes, 7.9 g lichens, 0.9 g ferns, 1.9 g cryptogams, and 506.0 g litter. This corresponds to overall average biomass across the twelve sites of 119.0, 100.0, 107.3 and 168.7 g/m^2^ for the three functional groups and litter, respectively. For an overview over the clean dataset see Table 2. The raw data is provided on the FunCaB OSF^27^, the code to download and clean the data can be found in the FunCaB GitHub repository^28^ in the file R/biomass/read_biomass.R.

### Dataset (ii): Species-level biomass in extra controls in 2016

This dataset contains the biomass removed per plot and species for forbs in the 48 extra control plots in 2016 (Tables 1, 3). Species-level biomass was recorded for 79 forb species, of which *Potentilla erecta* (24.3 g), *Achillea millefolium* (16.6 g), and *Alchemilla alpina* (16.3 g) were the most abundant overall. Abundances varied across sites, in the alpine *Alchemilla alpina* (12.9 g), *Alchemilla sp*. (10.0 g), and *Salix herbacea* (5.97 g) were the most abundant species, whereas in boreal sites *Achillea millefolium* (10.2 g), *Potentilla erecta* (6.88 g), and *Hieracium pilosella* (5.51 g), dominated. For an overview over the clean dataset see Table 3. The final cleaned data together with a data dictionary is provided on the FunCaB OSF^27^, and the code to download the final cleaned data can be found in the FunCaB GitHub repository^28^ in the file R/data_dic/download_clean_data.R.

**Table 3:** Data dictionary for the FunCaB species-level biomass (dataset ii).

Variable name	Description	Variable type	Variable range or levels	Units	How measured
year	Year of sampling	numeric	2016 – 2016	yyyy	defined
siteID	Unique site ID	categorical	Alrust – Vikesland		defined
blockID	Unique block ID a combination of site and block	categorical	Alr1 – Vik4		defined
plotID	Unique plot ID is a combination of site, block and treatment	categorical	Alr1XC – Vik4XC		defined
treatment	Plant functional groups removed, where F = forbs, G = graminoids, B = bryophytes and C = control, and XC = extra control	categorical	XC – XC		defined
functional_group	Removed functional group, including forbs, bryophytes, graminoids, litter, pteridophytes, lichens, and cryptograms	categorical	forb – forb		defined
species	Species abbreviation	categorical	Ach.mil – Vio.sp		defined
biomass	Dry weight of removed functional group	numeric	0.01 – 12.95	g	measured
sorted_by	Person that sorted the biomass	categorical	AP – PP		recorded

Data dictionary with column descriptions for dataset ii – the total forb biomass per species in the 48 extra controls in 2016 at twelve sites in the Vestland Climate Grid, Vestland county, Norway.

### Dataset (iii): Soil temperature

The iButton dataset contains soil temperature data per plot from July 2015 to July 2016 (Tables 1, 4). This dataset has data from 322 loggers with a total of 1,084,970 observations. Soil temperatures ranged from −8.25 to 32.8 °C. During the growing season months, May through August, the average daily mean temperature was lowest in plots where only forbs were present, 9.47 ± 0.16 °C (GB treatment) and highest in the bare ground plots 10.40 ± 0.14 °C (FGB treatment). During the same period, the daily max temperatures were also lowest where only forbs were present, 17.80 ± 0.16 °C (GB treatment) and highest in the bare ground plots 20.40 ± 0.14 °C (FGB treatment). For an overview over the clean dataset see Table 4. The final cleaned data together with a data dictionary is provided on the FunCaB OSF^27^, and the code to download the final cleaned data can be found in the FunCaB GitHub repository^28^ in the file R/data_dic/download_clean_data.R.

**Table 4:** Data dictionary for the FunCaB soil temperatures (dataset ii).

Variable name	Description	Variable type	Variable range or levels	Units	How measured
year	Year of sampling	numeric	2015 – 2016	yyyy	defined
date_time	Date and time of sampling (UTC + 2)	date_time	2015-07-12 17:58:01 – 2016-06-27 23:05:01	yyyy-mm-dd hh:mm:ss	defined
siteID	Unique site ID	categorical	Alrust – Vikesland		defined
blockID	Unique block ID a combination of site and block	categorical	Alr1 – Vik5		defined
plotID	Unique plot ID is a combination of site, block and treatment	categorical	Alr1B – Vik5GF		defined
iButtonID	Unique iButton ID	categorical	003E3B5C41_2016 – F83E3E2A41_2016		defined
treatment	Plant functional groups removed, where F = forbs, G = graminoids, B = bryophytes, and C = control	categorical	FGB, FG, FB, GB, G, F, B, C		defined
soiltemperature	Soil temperature measurement per plot	numeric	−8.75 – 32.8	°C	recorded
comments	Additional comments	categorical			
turfID	Unique turf ID from SeedClim transplant experiment	categorical	101 TTC – 73 TTC		defined

Data dictionary with column descriptions for dataset iii – soil temperature from 384 25 × 25 cm experimental plant functional group removal plots from 2015 – 2016 at twelve sites in the Vestland Climate Grid, Vestland county, Norway.

### Dataset (iv): Soil moisture

The soil moisture dataset contains volumetric soil moisture data per plot from June 2015 to August 2018 (Tables 1, 5). The database contains 4,771 soil moisture measurements from 2015 to 2019, with the median number of measurements per plot per year ranging from 3 – 5, except in 2018 where the median was one measurement per plot. Average soil moisture was 20.2 ± 0.4% in 2015, 26.1 ± 0.5% in 2016, 29.6 ± 0.6% in 2017, 19.0 ± 0.9% in 2018, and 13.7 ± 0.5% in 2019, which was a drought year. Plots with forbs only and all functional groups removed were the driest, with an average soil moisture of 21.8 ± 0.7% (F) and 22.1 ± 0.7 (FGB) whereas the intact control plots were the wettest with 24.1 ± 0.6% (C). For an overview over the clean dataset see Table 5. The final cleaned data together with a data dictionary is provided on the FunCaB OSF^27^, and the code to download the final cleaned data can be found in the FunCaB GitHub repository^28^ in the file R/data_dic/download_clean_data.R.

**Table 5:** Data dictionary for the FunCaB soil moisture (dataset iv).

Variable name	Description	Variable type	Variable range or levels	Units	How measured
date	Date of sampling	date	2015-06-02 – 2019-08-09	yyyy-mm-dd	defined
siteID	Unique site ID	categorical	Alrust – Vikesland		defined
blockID	Unique block ID a combination of site and block	categorical	Alr – Vik5		defined
plotID	Unique plot ID is a combination of site, block and treatment	categorical	Alr1B – Vik5GF		defined
treatment	Plant functional groups removed, where F = forbs, G = graminoids, B = bryophytes, and C = control	categorical	FGB, FG, FB, GB, G, F, B, C		defined
soilmoisture	Soil moisture measurement per plot	numeric	0 – 100	(m^3^ water × m^−3^ soil) × 100	recorded
weather	Weather conditions during data collection	categorical	Blue sky - windy, cloudy		recorded
recorder	Data collector	categorical	? – Vojta		recorded
turfID	Unique turf ID from SeedClim transplant experiment	categorical	101 TTC – TTC 281		defined

Data dictionary with column descriptions for dataset iv – soil moisture from 384 25 × 25 cm experimental plant functional group removal plots from 2015 – 2016 at twelve sites in the Vestland Climate Grid, Vestland county, Norway.

### Dataset (v): Vascular plant community composition and structure

**Fig. 5:**
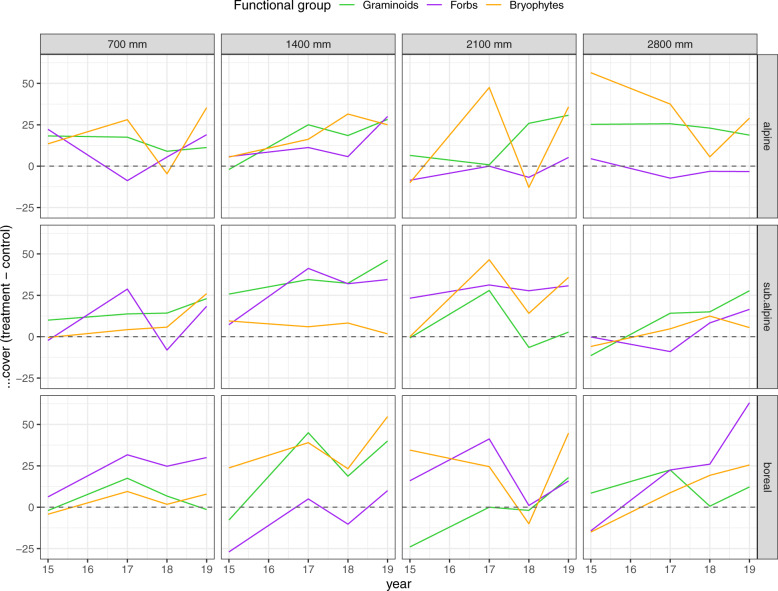
Functional group cover in response to removal treatments in the FunCaB plant functional group experiment. Difference from unmanipulated control plots (C treatment) over time in the sum of covers of graminoids, forbs, and bryophytes in plots where this functional group grows alone (i.e., the FB, GB, and GF removal treatments, respectively, see Table 6 for abbreviations). Data from 192 25 × 25 cm experimental plant functional group removal plots from 2015 – 2019 at twelve sites in the Vestland Climate Grid, Vestland County, Norway.

The plot-level plant community dataset contains a total of 206 taxa and 17,181 observations (taxa × plots × years) (Tables 2, 6). Overall, 299 observations (1.74%) were not identified to taxon. In 2015, the pre-treatment vegetation had an average species richness of 5.9 graminoids (ranging from 3.2 to 9.3 across sites) and 10.8 forbs (ranging from 3.8 to 14.9 across sites) per plot. The highest species richness was found in alpine sites. Sum of covers of the non-removed functional groups varied over sites and years (Fig. 5). In many cases, biomass of the remaining functional group increases over time in plots where other functional groups were removed, relative to controls, suggesting release from competitive interactions may be occurring. However, in some cases, notably forbs in colder and wetter sites, responses are neutral to negative, which is consistent with earlier work in the system suggesting that competitive plant-plant interactions are less important and facilitative interactions more important under these conditions^42^. Similarly, there were generally lower abundances and also lower relative biomass gains in removals in 2018, which was a drought year, suggesting competitive release was less important under this climate extreme event (Fig. 5). Note that some of the recorded data (e.g., sub-plot level presence data, life-history stage/fertility data, acrocarp/pleurocarp bryophyte cover data) are available in the raw data tables, but these are not processed or presented in the final data table. For an overview over the clean dataset see Table 6. The final cleaned data together with a data dictionary is provided on the FunCaB OSF^27^, and the code to download the final cleaned data and the taxon table can be found in the FunCaB GitHub repository^28^ in the file R/data_dic/download_clean_data.R.

**Table 6:** Data dictionary for the FunCaB plant community composition (dataset v).

Variable name	Description	Variable type	Variable range or levels	Units	How measured
year	Year of sampling	numeric	2015 – 2019	yyyy	defined
siteID	Unique site ID	categorical	Alrust – Vikesland		defined
blockID	Unique block ID a combination of site and block	categorical	Alr1 – Vik5		defined
plotID	Unique plot ID is a combination of site, block and treatment	categorical	Alr1B – Vik5GF		defined
removal	Pre (2015) or post ( >2015) removal treatment. Pre removal is related to the start of the experiment.	categorical	post – pre		defined
treatment	Plant functional groups removed, where F = forbs, G = Graminoids, and B = Bryophytes. C = Controls and XC = extra controls	categorical	FGB, FG, FB, GB, G, F, B, C, XC		defined
total_graminoids	Cover of graminoids	numeric	0 – 100	percentage	recorded
total_forbs	Cover of forbs	numeric	0 – 100	percentage	recorded
total_bryophytes	Cover of bryophytes	numeric	0 – 120	percentage	recorded
vegetation_height	Height of vegetation	numeric	0 – 350	mm	measured
moss_height	Height of bryophytes	numeric	0 – 120	mm	measured
litter	Cover of litter	numeric	1 – 100		recorded
species	Species abbreviation	categorical	Ach.mil – Vio.tri or NA		defined
cover	Individual species cover	numeric	1 – 98	percentage	recorded
functional_group	Removed functional group, including forbs, bryophytes, graminoids. For extra controls also litter, pteridophytes, lichens, and cryptograms	categorical	forb - graminoid		defined
sumcover	Total sum of species cover	numeric	1 – 176	percentage	recorded
recorder	Data collector	categorical	Aud – W		recorded
turfID	Unique turf ID from SeedClim transplant experiment	categorical	101 TTC – 73 TTC		defined

Data dictionary with column descriptions for dataset v – the plant community composition of 384 25 × 25 cm experimental plant functional group removal plots from 2015 – 2019, and in 48 extra controls in 2016, at twelve sites in the Vestland Climate Grid, Vestland County, Norway.

### Dataset (vi): Seedling recruitment

The seedling recruitment dataset has data on a total of 16,656 seedlings of 44 species (Tables 2, 7). Overall, 61.2% of the seedlings were identified to species, the remaining 38.8% were either unidentifiable for the duration of the study or they died before they could be identified. Seedling densities were highest in the bare soil and forb only plots (FGB and GB treatments) with 1,297 and 1,451 seedlings m^−2^, respectively, and lowest in the graminoid and bryophyte and graminoid only plots (F and FB treatments) with 228 and 224 seedlings m^−2^, respectively. The intact vegetation controls (C) had intermediate seedling densities, with 764 seedlings m^−2^. Seedling emergence differed between the two study years, with 5,888 seedlings recorded in the drought year 2018 compared to 10,768 in 2019. Overall, 5,870 seedlings survived till the last census. For an overview over the clean dataset see Table 7. The final cleaned data together with a data dictionary is provided on the FunCaB OSF^27^, and the code to download the final cleaned data can be found in the FunCaB GitHub repository^28^ in the file R/data_dic/download_clean_data.R.

**Table 7:** Data dictionary for the FunCaB seedling recruitment (dataset v).

Variable name	Description	Variable type	Variable range or levels	Units	How measured
year	Year of sampling	numeric	2018 – 2019	yyyy	defined
date	Date of sampling	date	2018-07-01 – 2019-08-15	yyyy-mm-dd	defined
siteID	Unique site ID	categorical	Alrust – Vikesland		defined
blockID	Unique block ID a combination of site and block	categorical	Alr1 – Vik5		defined
plotID	Unique plot ID is a combination of site, block and treatment	categorical	Alr1B – Vik5GF		defined
treatment	Plant functional groups removed, where F = forbs, G = graminoids, B = bryophytes and C = control, and XC = extra control	categorical	B – GF		defined
seedID	Unique seedling ID	categorical	1_Alr3B – z_Ves3GB		
round	Round of sampling	numeric	1 – 4	count	defined
species	Species abbreviation	categorical	Ach.mil – Vio.sp		defined
presence	Presence (1) or absence (0) of a seedling per census	numeric	0 – 1		recorded
x	x coordinate in the plot	numeric	0 – 250	mm	recorded
y	y coordinate in the plot	numeric	0 – 250	mm	recorded
comment	Comment on measurement	categorical	? – x220, y113		
turfID	Unique turf ID from SeedClim transplant experiment	categorical	11 TTC – 73 TTC		defined
functional_group	Removed functional group, including forbs, bryophytes, graminoids, litterm pteridophytes, lichens, and cryptograms	categorical	forb – forb		defined
round	Round of sampling; round 1–2 correspond to 2018, and round 3–4 to 2019	numeric	1 – 4		defined

Data dictionary with column descriptions for dataset v – the plant community composition of 384 25 × 25 cm experimental plant functional group removal plots from 2015 – 2019, and in 48 extra controls in 2016, at twelve sites in the Vestland Climate Grid, Vestland County, Norway.

### Dataset (vii)- Ecosystem carbon fluxes

The plot-level ecosystem carbon flux dataset has a total of 3,696 paired light (GPP) and dark (Reco) measurements between 2015 and 2017 (Tables 1, 8, 9). A total of 926 paired fluxes were recorded in 2015, 1,286 in 2016 and 1,484 in 2017. The median number of measurements per plot and year 2 in 2015, 8 in 2016 (when we measured control plots only) and 5 in 2017. The GPP values ranged from −24.30 ± 0.06 to −0.01 ± 0.06 µmol mol^−1^ s^−1^ CO_2_, the PAR between 4.66 ± 8.46 and 2555.00 ± 8.46 (µmol m^−2^ s^−1^), the soil moisture between 0 ± 0.28 and 76.78 ± 0.28 and the air temperature within the chamber varied between 6.08 ± 0.10 and 39.63 ± 0.10 °C. The respiration measurements (R_eco_) ranged from 0.55 ± 0.05 to 30.35 ± 0.05 µmol mol^−1^ s^−1^ CO_2_, the soil moisture between 0 ± 0.28 and 76.78 ± 0.28 and the associated air temperature varied within the chamber measurements between 6.08 ± 0.10 and 38.83 ± 0.10 °C. Note that light and dark measurements have very similar soil moisture and air temperature, as would be expected given that they are both measured on the same plots within a short period. Average GPP varied among treatments, and on average, the highest CO_2_ uptake was found in intact vegetation (C; −8.28 ± 0.19 µmol mol^−1^ s^−1^ CO_2_) while the lowest uptake was found in bare ground plots (FGB treatment; −4.95 ± 0.27 µmol mol^−1^ s^−1^ CO_2_). Respiration did not vary much across treatments, but for GPP, the highest value 6.63 ± 0.15µmol mol^−1^ s^−1^ CO_2_ was again found in the intact vegetation (C) and the lowest value 5.04 ± 0.17 µmol mol^−1^ s^−1^ CO_2_ in the bare ground plots (FGB treatment). For an overview over the clean dataset see Tables 8, 9. The final cleaned data together with a data dictionary is provided on the FunCaB OSF^27^, and the code to download the final cleaned data can be found in the FunCaB GitHub repository^28^ in the file R/data_dic/download_clean_data.R.

**Table 8:** Data dictionary for the FunCaB ecosystem light measurement carbon fluxes (dataset vii).

Variable name	Description	Variable type	Variable range or levels	Units	How measured
year	Year of sampling	numeric	2015 – 2017	yyyy	defined
date	Date of sampling	date	2015-06-30 – 2017-08-01	yyyy-mm-dd	defined
siteID	Unique site ID	categorical	Alrust – Vikesland		defined
blockID	Unique block ID a combination of site and block	categorical	Alr1 – Vik5		defined
plotID	Unique plot ID is a combination of site, block and treatment	categorical	Alr1B – Vik5RTC		defined
treatment	Plant functional groups removed, where F = forbs, G = graminoids, B = bryophytes, C = control, XC = extra control, RTC = VCG graminoid removal	categorical	FGB, FG, FB, GB, G, F, B, C, XC, RTC		defined
starttime	Start time of light measurement (UTC + 2)	date_time	2015-06-30 08:10:30 – 2017-08-01 14:17:10	yyyy-mm-dd hh:mm:ss	recorded
stoptime	End time of light measurement (UTC + 2)	date_time	2015-06-30 08:12:30 – 2017-08-01 14:19:10	yyyy-mm-dd hh:mm:ss	recorded
PAR	PAR value of light measurement	numeric	4.659 – 2555	µmol m–2 s–1	recorded
soiltemp	Soil temperature value of light measurement	numeric	3 – 23.2	°C	recorded
soilmoisture	Soil moisture measurement per plot	numeric	0 – 76.775	m3 water×m–3 soil×100	recorded
tempK	Air temperature in the chamber during light measurement	numeric	279.225 – 312.784	Kelvin	recorded
vegHeight	Vegetation height	numeric	0.01 – 27	mm	measured
nee	Net ecosystem exchange	numeric	−15.392 – 17.885	µmol m^−2^ s^−1^	calculated
rsqd	R squared of slope of linear regression fitting the CO2 concentration versus time	numeric	0 – 1		calculated
chamber	Carbon flux chamber	numeric	1 – 2		defined
removal	Pre (2015) or post (>2015) removal treatment. Pre removal is related to the start of the experiment.	categorical	post – pre		defined
weather	Weather conditions during data collection	categorical	cloud – Windy		recorded
flag	Flag for data quality (x or DROP)	categorical			
comment	Comment on measurement	categorical			
time	Time in seconds for light measurement	numeric	0 – 195	second	recorded

Data dictionary with column descriptions for dataset vii – the ecosystem light measurement carbon fluxes from 384 25 × 25 cm experimental plant functional group removal plots from 2015 – 2017, and in 48 extra controls in 2016, at twelve sites in the Vestland Climate Grid, Vestland County, Norway. Note that the dataset also contains additional measurements from another graminoid removal experiment in the VCD and the corresponding dark measurements for each plot and measurement time (Table 9).

**Table 9:** Data dictionary for the FunCaB ecosystem dark measurement carbon fluxes (dataset vii).

Variable name	Description	Variable type	Variable range or levels	Units	How measured
year	Year of sampling	numeric	2015 – 2017	yyyy	defined
date	Date of sampling	date	2015-06-30 - 2017 – 08-01	yyyy-mm-dd	defined
siteID	Unique site ID	categorical	Alrust – Vikesland		defined
blockID	Unique block ID a combination of site and block	categorical	Alr1 – Vik5		defined
plotID	Unique plot ID is a combination of site, block and treatment	categorical	Alr1B – Vik5RTC		defined
treatment	Plant functional groups removed, where F = forbs, G = graminoids, B = bryophytes, C = control, XC = extra control, RTC = VCG graminoid removal	categorical	FGB, FG, FB, GB, G, F, B, C, XC, RTC		defined
vegHeight	Vegetation height	numeric	0.01 – 27	mm	measured
gpp	Gross primary production calculated from nee - Reco	numeric	−24.342 – −0.004	µmol m^−2^ s^−1^	calculated
starttime_Reco	Start time of dark measurement (UTC + 2)	date_time	2015-06-30 08:14:30 – 2017-08-01 14:19:55	yyyy-mm-dd hh:mm:ss	recorded
stoptime_Reco	End time of dark measurement (UTC + 2)	date_time	2015-06-30 08:16:00 – 2017-08-01 14:21:55	yyyy-mm-dd hh:mm:ss	recorded
time_Reco	Time in seconds for dark measurement	numeric	5 – 180	second	recorded
PAR_Reco	PAR value of dark measurement	numeric	0 – 140.131	µmol m^−2^ s^−1^	recorded
soiltemp_Reco	Soil temperature value of dark measurement	numeric	3 – 23.2	°C	recorded
tempK_Reco	Air temperature in the chamber during dark measurement	numeric	279.225 – 311.977	Kelvin	recorded
Reco	Ecosystem respiration	numeric	0.549 – 30.354	µmol m^−2^ s^−1^	recorded
rsqd_Reco	R squared of slope of linear regression fitting the CO2 concentration versus time	numeric	0.8 – 1		calculated
chamber_Reco	Carbon flux chamber	numeric	1 – 3		defined
delta	Time difference between dark and light measurement	numeric	65 – 7050	second	calculated
removal	Pre (2015) or post (>2015) removal treatment. Pre removal is related to the start of the experiment.	categorical	post – pre		defined
weather	Weather conditions during data collection	categorical	cloud – Windy		recorded
flag	Flag for data quality (x or DROP)	categorical			
comment	Comment on measurement	categorical			
time	Time in seconds for light measurement	numeric	0 – 195	second	recorded

Data dictionary with column descriptions for dataset vii – the ecosystem dark measurement carbon fluxes from 384 25 × 25 cm experimental plant functional group removal plots from 2015 – 2017, and in 48 extra controls in 2016, at twelve sites in the Vestland Climate Grid, Vestland County, Norway. Note that the dataset also contains additional measurements from another graminoid removal experiment in the VCD and the corresponding light measurements for each plot and measurement time (Table 8). The light measurement data are used to calculate gpp (gross primary production) and delta (time difference between light and dark measurements).

### Dataset (xi) - Reflectance

The plot-level community reflectance dataset has a total of 1,244 observations. In 2019 we made 480 post-treatment measurements, and in 2021 we made 382 pre-treatment measurements and 382 post-treatment measurements (Tables 2, 10). Average reflectance post-treatment was 0.634 ± 0.004 (mean ± SE) in 2019 and 0.679 ± 0.006 in 2021, whereas the reflectance pre-treatment in 2021 was 0.760 ± 0.010. In both years, the highest reflectance post cut was found in the intact vegetation (C), 0.695 ± 0.007 in 2019 and 0.774 ± 0.009 in 2021, whereas the lowest reflectance was found in the bare ground treatment (FGB) 0.524 ± 0.008 in 2019 and 0.507 ± 0.015 in 2021. For an overview over the clean dataset see Table 10. The final cleaned data together with a data dictionary is provided on the FunCaB OSF^27^, and the code to download the final cleaned data can be found in the FunCaB GitHub repository^28^ in the file R/data_dic/download_clean_data.R.

**Table 10:** Data dictionary for the FunCaB vegetation reflectance (dataset viii).

Variable name	Description	Variable type	Variable range or levels	Units	How measured
date	Date of sampling	date	2019-07-19 – 2021-07-30	yyyy-mm-dd	defined
siteID	Unique site ID	categorical	Alrust – Vikesland		defined
blockID	Unique block ID a combination of site and block	categorical	Alr1 – Vik5		defined
plotID	Unique plot ID is a combination of site, block and treatment	categorical	Alr1B – Vik5GF		defined
treatment	Plant functional groups removed, where F = forbs, G = graminoids, B = bryophytes and C = control, extra controls	categorical	FGB, FG, FB, GB, G, F, B, C, XC		defined
pre_post_cut	Measurement was taken before or after the cut	categorical	post – pre		recorded
ndvi	NDVI value	numeric	0.315 – 4.265	percentage	measured
notes	Notes	categorical			
turfID	Unique turf ID from SeedClim transplant experiment	categorical	TTC 101 – TTC 69		defined
weather	Weather conditions during data collection	categorical	Cloudy – Sunny		recorded
time	Time of sampling (UTC + 2)	time	08:00 – 19:55	hh:mm	defined

Data dictionary with column descriptions for dataset vii – the vegetation reflectance from 384 25 × 25 cm experimental plant functional group removal plots from 2019 and 2021, at twelve sites in the Vestland Climate Grid, Vestland County, Norway.

## Technical Validation

### Experimental validation

The plant functional group experiments and field measurements (before-after treatment photographs, soil moisture measurements, site-level VCD climate data) were maintained over seven years under the oversight of LCK, supported by IHJA and SAHÖ in some years and field campaigns. Consistent leadership of the campaigns ensured consistency in experimental treatments and data collection, which was critical as a large number of students, technicians and interns were involved in the plant functional group removal work, typically 4 – 6 people each year (see Acknowledgements). Errors made during field work are noted in the data sheets and entered as comments in the plant functional group removal biomass data tables. The photographs of each plot pre- and post-removal for each field campaign (with some gaps, and photos are lacking from 2020) can be used for further documentation and validation^27^. Biomass removed decreased over time, especially after the second year (Fig. 5, suggesting the communities stabilised in the experimentally defined functional group composition, with relatively little regrowth of the removed functional group after this point.

### Taxonomic validation

During the 7-year data collection period a number of people were involved in community and seedling data collection (see notes in dataset for recorder information), which introduces a risk of observer errors. In particular, species can be misidentified (e.g., sterile graminoids) or might be overlooked in one of the community censuses. Such errors, if uncorrected, generate pseudo-turnover in the plant community and seedling data. To detect and correct errors in the community data, we compared each recorded species in each plot or subplot over the time-series. We used these comparisons to assign unidentified or missing species to an existing identified species in that plot if it was likely that this could be the correct ID, and if there was a record of that species in the plot in the previous and following year. Further, we imputed species covers in cases where cover was either missing or clearly too low or high to be realistic when comparing with the total sum of covers and covers of the same species in adjacent years in the time-series, replacing such erroneous values with the mean cover from the previous and following year. We further checked and corrected ‘botanist effects’ in cover estimation between observers, in height estimations (using cm vs mm), and ensured taxonomic consistency. The data-checking code and outcomes for these various procedures is documented in^28^. In addition, 23 forbs and six graminoid taxa are identified to the genus level in the community dataset and one graminoid, two forbs and seven seedling observations are unidentified (these are named NID.herb, NID.gram, and NID.seedling in the datasets)(datasets v, vi). A full species list of all identified taxa across datasets is also available in the taxon table in the OSF repository (see Table 2). This table also includes the functional group (F, G, or B) to which each taxon was assigned in the experimental treatments. Note that the sub-plot-level presences and life-history stage/fertility data are available in the OSF, but these data have not been processed or cleaned.

### Ecosystem C flux validation

All measurements were first visually evaluated for data quality. If there were clear outliers (values far outside the normal range of CO_2_ concentrations within a measurement), these values were flagged and removed before flux calculation. The timeframes of measurements were adjusted if the linear relationship over time was affected by either change in cloud cover or there was indication of chamber leakage (CO_2_ increase/decrease levelling off after steady linear relationship). Resulting fluxes were only included if they showed a consistent linear relationship between CO_2_ and time for a period of at least 60 seconds. Measurements of poor quality due to erratic CO_2_ concentrations over time were flagged and removed. An additional criterion was that this relationship had R^2^  ≤ 0.2 or R^2^ ≥ 0.8 for NEE measurement in light conditions and R^2^ ≥ 0.8 for NEE dark measurements (R_eco_; 52, 150 and 466 measurements removed in 2015, 2016 and 2017, respectively). Measurements of NEE in light conditions with R^2^ ≤ 0.2 ensure inclusion of measurements with equal rates for R_eco_ and GPP. Dark measurements with resulting NEE < 0 were removed, as they would indicate uptake CO_2_ uptake (49 measurements removed in 2017). Sets of L and D measurement per plot were then matched together based on date and time of day for calculation of GPP but were not paired if they were more than 2 hours apart from each other. Finally, GPP fluxes with values > 0 were removed, as they would represent CO_2_ release, not uptake/primary productivity (0 measurements were thus removed in 2015, 18 in 2016, and 191 in 2017). The PAR sensor for one of the chambers was malfunctioning between 03 July 2017 and 6 July 2017. PAR values for these measurements were corrected by using an estimated value based on recorded PAR values in the metadata from the other par sensor.

## Usage Notes

### Data use best practice

We suggest that all FunCaB project data presented here and available in the FunCaB OSF^27^ be cited to this data paper. Other VCG data available through the VCG OSF^35^ should be cited to the relevant publications, specifically, the general description of the climate and environment, soils, land-use and environment, vegetation, and ecosystems along with basic descriptive data of the 12 sites are presented in^34^, whereas specific biotic and abiotic response data are presented in e.g.^37–48^,. Note that major VCG datasets will be made available in a forthcoming data paper, and they can henceforth be cited to that paper. See Fig. 3 for a description of the interrelations and linkage keys among datasets.

The data are available for use under a CC-BY licence. We appreciate being contacted for advice or collaboration, if relevant, by users of our data. In cases where our data make up >10% of the data used in a downstream publication, we expect that appropriately acknowledging our contributions would require an invitation for collaboration.

### Combination and augmentation with existing and new data from the VCG

We note that for plant functional group removal biomass (dataset i), soil moisture (dataset iv), plant community (dataset v), carbon flux (dataset vii) and reflectance (dataset viii), a final census planned for 2022. These data will be added to^27^ and^28^.

The data presented here relates to a large amount of site and block level data from the same sites^35^, the most relevant of which are briefly explained under ‘Background abiotic and biotic data from the Vestland Climate Grid’ above. Code is provided for linking and downloading the most relevant datasets in the FunCaB github repository, with a time-stamper version on Zenodo^28^. See Fig. 2 for a conceptual representation of how these datasets are linked via shared variables/keys.

In connection with a new project, FUNDER (Norwegian Research Council project number 315249, 2021 – 2025), the FunCaB experiment will be augmented with data on the belowground components of the plant-soil ecosystem, including roots, mesofauna, fungi and microbes. Plot-level slope, aspect, and soil depth will also be measured. These data will link with the FunCaB and VCG project based on the given experimental, site and organismal keys, as indicated in Fig. 3.

The VCG and FUNDER data will be made available in forthcoming data papers.

### Data quality comments and options

The procedures and consequences for data collection, management, and cleaning are detailed in this paper, and the associated code^28^. The code^28^ describes and implements our suggested data cleaning and checking procedures that result in producing what we consider the clean and ‘best practice’ final datasets^27^ and the various ‘flag’, ‘comment’ or’notes’ columns in the different dataset tables (Tables 2–9) that indicate additional specific data points that could be removed to create even more robust datasets. Users who might prefer stricter or more inclusive data handling strategies should check the flags in the raw data sets and adjust the data cleaning accordingly.

Note that unidentified taxa are not harmonized across datasets (relevant for datasets ii, v, vi; Table 1).

Also note that dataset ii species-level forb biomass has some blocks where data at the species level is lacking (11 cases, out of 48). For these blocks, the forb biomass is provided at the functional group level.

Dataset vi, seedling recruitment, does not contain any information on the plots that did not have any seedlings. If the user wants a complete dataset with all plots per site, for example for calculating overall seedling densities per treatment or site, these missing plots need to be extracted from another dataset, e.g. dataset v, plant community composition.

For dataset vii, ecosystem carbon fluxes, users might want to set more or less restrictive data exclusion thresholds regarding linear fits of fluxes to include in analysis, and can do so by altering the cleaning code. For example, many paired light-dark measurements were deleted due to the quality control of the light measurements. Users who are interested in dark measurements only might be interested in accessing the full dark measurements dataset available from the raw data available in^27^. If users want to visually inspect individual measurements and set their own timeframes the code for running through individual combinations of metadata, logger data and temperature data are provided in the OSF. Note that in 2016, some control plots shared with another experiment (treatment = TTC) and other graminoid removal plots (treatment = RTC) from another project^35^, see also^42^, were also measured. These measurements are found in the FunCaB flux dataset.

## Data Availability

The code used for checking, cleaning and analysing the data is available in the open GitHub repository https://github.com/Between-the-Fjords/funcab_data, of which a versioned copy is available at Zenodo^54^.
